# Supplementary material for: A fault-intrusion-tolerant system and deadline-aware algorithm for scheduling scientific workflow in the cloud
Source: PeerJ Comput Sci. 2021 Nov 2;7:e747. doi: 10.7717/peerj-cs.747 (PMC8576554; doi:10.7717/peerj-cs.747)
Supplement: Supplemental Information 6 [file peerj-cs-07-747-s006.zip › Myproject1.1/docs/org/workflowsim/utils/package-summary.html]

org.workflowsim.utils


JavaScript is disabled on your browser.


- Overview
- Package
- Class
- Use
- Tree
- Deprecated
- Index
- Help

- Prev Package
- Next Package

- Frames
- No Frames

- All Classes

# Package org.workflowsim.utils

- Class Summary

  | Class | Description |
  | --- | --- |
  |  |  |
  | --- | --- |
  | ArgumentParser | This class implements ArgumentParser that parse arguments and set the parameters |
  | ClusteringParameters | ClusteringParameters contains all the parameters used in task clustering |
  | OverheadParameters | This class includes all parameters that involve overheads |
  | Parameters | This class includes most parameters a user can specify in a configuration file |
  | ReplicaCatalog | ReplicaCatalog stores all the data information and where (site) there are |
- Enum Summary

  | Enum | Description |
  | --- | --- |
  |  |  |
  | --- | --- |
  | ClusteringParameters.ClusteringMethod | Supported Clustering Method, by default it is none |
  | Parameters.FTCFailure |  |
  | Parameters.FTCMethod | Fault Tolerant Clustering method |
  | Parameters.FTCMonitor |  |
  | Parameters.PLNMethod | Planning Algorithm (Global Scheduling Algorithm) |
  | Parameters.SCHMethod |  |
  | ReplicaCatalog.FileSystem | File System |

- Overview
- Package
- Class
- Use
- Tree
- Deprecated
- Index
- Help

- Prev Package
- Next Package

- Frames
- No Frames

- All Classes
